# Addressing NCDs through research and capacity building in LMICs: lessons learned from tobacco control

**DOI:** 10.3402/gha.v9.32407

**Published:** 2016-08-19

**Authors:** Rachel Sturke, Susan Vorkoper, Kalina Duncan, Marya Levintova, Mark Parascondola

**Affiliations:** 1 Fogarty International Center, US National Institutes of Health, Bethesda, MD, USA; 2 National Cancer Institute, US National Institutes of Health, Bethesda, MD, USA

**Keywords:** non-communicable diseases, research capacity, capacity building, tobacco control, scientific evidence, global health

## Abstract

Confronting the global non-communicable diseases (NCDs) crisis requires a critical mass of scientists who are well versed in regional health problems and understand the cultural, social, economic, and political contexts that influence the effectiveness of interventions. Investments in global NCD research must be accompanied by contributions to local research capacity. The National Institutes of Health (NIH) and the Fogarty International Center have a long-standing commitment to supporting research capacity building and addressing the growing burden of NCDs in low- and middle-income countries. One program in particular, the NIH International Tobacco and Health Research and Capacity Building Program (TOBAC program), offers an important model for conducting research and building research capacity simultaneously. This article describes the lessons learned from this unique funding model and demonstrates how a relatively modest investment can make important contributions to scientific evidence and capacity building that could inform ongoing and future efforts to tackle the global burden of NCDs.

## Introduction

An essential step to combating the rapidly rising burden of non-communicable diseases (NCDs) in low- and middle-income countries (LMICs) is to build local capacity to address the epidemic's evidence needs. Of the 52.8 million deaths in 2010, NCDs accounted for 34.5 million and nearly 75% of those now occur in LMICs (1). In 2013, the World Health Organization (WHO) endorsed the Global Action Plan for the Prevention and Control of NCDs (2013–2020) that promotes and supports national capacity for high-quality research and health system development (2). This plan responds, in part, to the incongruity between the rising burden of NCDs and adequate research evidence and capacity to address this challenge in LMICs, noting that country demands for conducting research on prevention and control of NCDs and the capacity to respond to those demands are not aligned (3). Indeed, investments in global NCD and related risk factors research must be accompanied by contributions to local research capacity. Addressing the WHO goals and confronting the global NCD crisis will require both new scientific evidence and a critical mass of scientists who are well versed in both regional health problems and understand the cultural, social, economic, and political contexts that influence patterns of disease and the effectiveness of interventions. To achieve this goal, we can look to innovative and proven approaches that simultaneously build research capacity and support research.

In every region of the world, the Fogarty International Center advances the National Institutes of Health (NIH) mission by supporting and facilitating global health research conducted by the United States and international investigators, building partnerships between health research institutions in the United States and abroad, and training the next generation of scientists to address global health needs. Fogarty and the NIH have a long-standing commitment to supporting research capacity building and addressing the growing burden of NCDs in LMICs through various programs (see Appendix A for a list of select Fogarty grant programs that support NCD research and capacity building). One program in particular, the NIH International Tobacco and Health Research and Capacity Building Program (TOBAC program), offers an important model for conducting research and building research capacity simultaneously and could inform ongoing and future efforts to tackle the burden of NCDs. Over the past decade, the TOBAC program has sought to address the challenges of tobacco use and associated disease and disability by funding collaborative research and capacity building projects. Using a unique funding model that supports research capacity building and the generation of empirical evidence simultaneously, the TOBAC program has successfully supported scientific advances that are moving the field forward, building capacity in epidemiological and behavioral research, prevention, treatment, communications, implementation, health services and policy research, and building sustainable partnerships, networks, and collaborations (4).

The goal of this article is to describe lessons learned from the TOBAC program that can inform future efforts to address the global challenge of NCDs.

## The TOBAC program

Tobacco use as a leading cause of preventable death and disease worldwide is responsible for almost 6 million deaths annually, accounting for 71% of lung cancers, 42% of chronic obstructive pulmonary disease, and nearly 10% of cardiovascular disease cases (5). However, while tobacco use has seen a steady decline in most high-income countries (HICs), in part due to important, evidence-based interventions, most LMICs have not seen the same progress (6). A disproportionate share of the global tobacco burden falls on LMICs, where 84% of the world's 1.3 billion current smokers reside (7). Decades of research in tobacco control have contributed to the evidence-base for the Framework Convention on Tobacco Control (FCTC), the first-ever international treaty negotiated under the auspices of the WHO, that aims to address the causes of the global tobacco epidemic, and the WHO MPOWER framework, a package of six evidence-based tobacco control measures that correspond to parts of the FCTC. Both are widely recognized as crucial forces spurring the adoption of evidence-based tobacco control policies around the world. Organizations funding research, like the International Development Research Centre (Canada) and the Rockefeller Foundation (US), have complimented efforts at Fogarty to support and advance international tobacco control research and capacity. Additional efforts of philanthropic organizations, such as the Bill and Melinda Gates Foundation and the Bloomberg Philanthropies, have made a substantial contribution through supporting tobacco control advocacy efforts in LMICs. The Council on Foreign Relations, an independent, nonpartisan membership organization and think tank and trusted source of information on global issues, recently published a report on the emerging global health crisis of NCDs and identified US Government leadership and investments in tobacco control as an action that would have substantial payoffs for global morbidity and mortality (8). Efforts to support long-term, sustainable NCD programs in LMICs will require ongoing research efforts and the development of research capacity in LMICs.

**Table:** 

Key structural characteristics of the TOBAC program
**True partnership between HIC and LMIC researchers**. Promoting international cooperation between investigators in HICs and LMICs ensures a two-way learning experience. At the same time, the majority of funds and research activity take place at the LMIC institution.
**Requirement for both research and capacity building that strengthens the ability to conduct research together with generating evidence**. By funding empirical research and capacity building simultaneously, TOBAC strengthens research capacity in concert with generating evidence to inform the evolving tobacco control research landscape.
**Condition that research be locally relevant and address a need of the host country/countries**. The TOBAC program enhances the number and knowledge of tobacco investigators and their capacity to conduct rigorous tobacco-related research essential to a country's ability to implement and evaluate tobacco control programs and policies.

Established in 2002, the NIH TOBAC program led by the Fogarty International Center in collaboration with NIH partners, including the National Cancer Institute and the National Institute on Drug Abuse, responds to this challenge and funds collaborative research and capacity building projects that address the burden of tobacco use in LMICs. The TOBAC program has two overarching goals: 1) pursue observational, intervention and policy research of LMIC relevance and 2) build capacity in epidemiological and behavioral research, prevention, treatment, communications, implementation, health services, and policy research. Tobacco control research and research capacity, in particular, are crucial to reducing tobacco use and related diseases; therefore, the TOBAC program is structured to make a significant contribution to research capacity while simultaneously supporting the generation of research evidence (see key characteristics of the TOBAC program). To date, 30 unique projects have been funded over three rounds in 2002, 2007, and 2012 (see Appendix B for a full list of TOBAC research projects). Over the first 11 years of the TOBAC program, projects received a total of $40.7M with an average award size of $307,000 per year. The results of this work, as described in a 10-year program review, include advances in tobacco control science and research capacity, the development of a global community of tobacco control researchers, as well as an enhanced understanding of models for evidence informing policy and practice (4).

Indeed, the success of the TOBAC-funded projects demonstrates the ability of a relatively small number of grants to advance tobacco control efforts on an international scale. Replicating these evidence and research capacity efforts for NCDs more broadly could go a long way toward addressing the burgeoning global burden of NCDs.

## Research capacity building

Today's complex NCD challenges require scientific competence nationally and internationally and a cadre of researchers with appropriate research training and support who can conduct research in LMICs. As a WHO report defines it, research capacity strengthening ‘includes any efforts to increase the ability of individuals and institutions to undertake high-quality research and to engage with the wider community of stakeholders (9)’. Capacity building activities not only support LMIC researchers and organizations, they also provide opportunities for investigators from HICs to gain knowledge of and experience in NCD issues in LMICs (10). In addition to training individual researchers, building capacity ultimately strengthens institutional capacity and can help create a culture of science at academic institutions.

Addressing NCDs necessitates a critical mass of highly skilled scientists who understand both regional health problems and the cultural, social, economic, and political contexts that influence the effectiveness of health interventions. Robust research capacity will enable researchers to more effectively address the global challenges of NCDs and enhance the use of scientific evidence in policy and practice. Furthermore, strengthening local capacity increases sustainability of these policies and programs. Local researchers and institutions are well-positioned to respond to the local context and changes in the NCD landscape over time by generating new, relevant knowledge to inform modifications to or development of novel approaches to addressing this burden. Fogarty has demonstrated this commitment to capacity building with more than four decades of support and funding of research-related capacity building activities, resulting in the training of thousands of scientists poised to make scientific discoveries in global health in more than 100 countries and in collaborations with more than 230 US and LMIC research institutions.

The TOBAC program, which requires capacity building as a primary goal alongside research, illustrates a novel model that has resulted in important capacity-related outcomes. Over the course of its first 10 years, TOBAC-funded projects trained more than 3,500 individuals (4). Of these individuals, more than 1,000 were long-term trainees educated in tobacco control research. TOBAC-funded training activities include training in areas ranging from tobacco control epidemiology, to research design, to strategies for enhancing the use of evidence to inform policy and program. For example, one grantee built and trained epidemiological teams to conduct morbidity and mortality studies on tobacco use for over 2 million adults in India (11). Other training programs focused on building research capacity to increase the expertise of researchers across the spectrum of tobacco control in regions of great need, such as sub-Saharan Africa and the Middle East (4). These efforts have resulted in a cadre of scientists in foreign institutions who are capable of addressing the complex global health challenge of tobacco control.

The TOBAC program also supports capacity strengthening at the institutional level. As centers for scientific discovery and training of future leaders, academic institutions play a unique role in stimulating researchers and key stakeholders to address priority health challenges, like tobacco control. The TOBAC program supports the development of robust partnerships between the United States and LMIC universities and research institutions, collaborations that have been the backbone of Fogarty's programmatic investments and that are an essential ingredient to building sustainable institutional research capacity. Strong collaborations enable institutions to leverage their strengths and advance the development of priority interventions for global health (see case study below). Supporting collaborative interactions encourages information sharing and ultimately the development of relationships between research institutions that serve as centers of excellence and regional resources for training and research. Such collaborations generate critical scientific information that can ultimately inform policies and programs aimed at reducing the consequences of tobacco consumption.

TOBAC grantees have employed a variety of collaboration-building models from technical and research exchanges, to more formal partnership among researchers, to strengthening relationships with implementing agencies as a means to ensure that evidence informs program implementation. By the 2007 funding cycle, TOBAC grantees had reported more than 140 new partnerships and collaborations as a result of the program (4). In one example in Southeast Asia, a grantee organized a network and workshops to address the challenges of identifying smuggled and counterfeit cigarettes (12). This effort convened policy makers and researchers from various countries in the region to discuss the difficulty of identifying smuggled and counterfeit cigarettes, shared data about the country-level techniques for marking cigarettes, and discussed plans for future work in this area. In countries around the world, TOBAC grantees’ capacity building and infrastructure activities have increased institutional research capacity across diverse organizations engaged in tobacco control research. Moreover, as the LMIC institutions have gained experience in grants management and collaboration, they are able to become more competitive for future research support thereby ensuring that their work continues.

**Table:** 

Case study: increasing capacity at the Mexican National Institute for public health
In Mexico, a grantee working with the National Institute for Public Health helped initiate a capacity building program in tobacco control research. The core capacity building elements were in-depth trainings for tobacco control researchers throughout the Latin America and the Caribbean, and educational opportunities for public health and biostatistics students attending the United States institution. The group conducted regional training workshops to establish a network of tobacco control experts to develop collaborative research projects. This led to policy-relevant epidemiologic and intervention research to estimate medical costs associated with tobacco use, income expenditures on tobacco, and surveillance of point of purchase marketing. As a result, the Mexican National Institute for Public Health has a well-established and locally sustained research program that plays a critical role in tobacco control nationally and provides regional training and coordination among national tobacco control researchers (13, 14).

## Enhanced empirical evidence

Although there is a growing body of research evidence to address the burden of NCDs, the evidence comes largely from HICs. For example, the bulk of the evidence for successful interventions, like smoking cessation, is from HICs, which may not directly translate to low-resource environments or settings where people have limited interaction with the health system (15). Indeed there are a substantial number of countries still lacking adequate data on NCDs and their related health outcomes (16). Additionally, local policy makers and government ministries want to see existing evidence adapted to their own national context. To achieve this, there is often a need for locally relevant research that demonstrates the overall health and economic effects of interventions that address NCDs. Important examples of this can be found in the TOBAC program. A TOBAC project working in Syria and Lebanon focused on understanding addiction and smoking behavior in waterpipe users (17). While a large body of evidence exists on cigarette smoking and nicotine addiction, including effective treatment strategies, limited data exist about long-term waterpipe users (18). In regions where waterpipe use is prevalent, and growing among younger people, this research conducted by a TOBAC grantee addressed a key gap.

While valuing capacity building, the TOBAC program simultaneously supports the generation of scientific evidence and also recognizes the importance of translating evidence to policy. In fact, the TOBAC program has resulted in significant scientific advances that have informed the implementation of FCTC objectives, enhanced knowledge of tobacco control in LMICs, and informed important policy and practice outcomes (4). TOBAC projects have contributed to progress in almost every FCTC article by generating evidence in epidemiological, behavioral, risk factor, intervention, cessation, policy, and economic research areas as demonstrated in the more than 400 articles published with TOBAC support (4).

In addition to contributing to the growing scientific body of evidence in LMICs, data generated by TOBAC-funded researchers have provided a critical evidence-base that has successfully informed international and national tobacco control policies. Grantees have been active in disseminating research results outside the scientific community. Examples of these policies have emerged from the work of a TOBAC grantee in Hungary and Romania whose work training scientists and health professionals to both conduct and disseminate research contributed to changes in national tobacco control policy (see case study below).

Similarly, TOBAC researchers have studied barriers to effective implementation of tobacco control measures. Understanding these barriers is critical to the uptake of evidence into policy and practice. For example, a TOBAC grantee looked at the effects of a 2009 tobacco tax increase in China that did not result in significant changes in the market (19). In this case, an increase in the excise tax on cigarettes raised additional revenue from producers but did not lead to an increase in the retail price for consumers. Thus, the tax did not have an impact on tobacco consumption or purchasing behavior (20). Using an innovative approach that brought together health experts and economists to conduct research on tobacco taxation policy, the project grappled with the important impact of tobacco tax adjustment.

**Table:** 

Case study: translating research to policy in Hungary
In Hungary, a country with some of the highest smoking rates in Europe, local TOBAC investigators focused on opportunities to relay scientific findings through educational presentations to legal and public health officers of local governments. Subsequently, tobacco sales tax increased nine times over from 2007 to 2011, an example of how evidence and data can help inform sound national health policy. Around the same time, the State Secretary's cabinet passed national clean air laws to protect non-smokers in public places, such as workplaces and restaurants. More recently, the team has been working in Romania to develop policy-relevant research capacity. The research team provided data to policy makers during discussions in Fall 2015 to strengthen the national smoke-free law. In December, a new law was passed in Parliament to expand smoke-free spaces to include bars and playgrounds (21).
One of the aims of the Hungary and Romania TOBAC program is training scientists, public health officials, NGO representatives, and others to take the lead in national tobacco control planning. This focus helped Hungarian and Romanian scientists not only become research experts in tobacco control science, it also encouraged them to understand the appropriate data needed to inform lawmakers about the harmful impacts of tobacco use (22–24).

## Key challenges and lessons learned for NCD research and capacity building

There is consensus among research funders that LMIC researchers are best placed to identify and address the health challenges of their own nations and to provide local and national policy makers with a broad range of high-quality, relevant evidence to inform decision-making (9). Worldwide, the highest burden of disease comes from LMICs, yet health research originating from these countries is low. According to one study, sub-Saharan Africa contributes to less than 1% of global biomedical publications (25). Strengthening research capacity in LMICs is the only way to address global health challenges and is one of the prerequisites to meeting development goals (9). The TOBAC program offers a useful model for supporting the generation of scientific evidence while simultaneously strengthening research capacity. Indeed, a review of the first 10 years of the TOBAC program demonstrates the success of this model and the progress made in tobacco control research and research capacity building. With NCDs expected to rise in LMICs and the resulting health epidemics, there is a critical need to learn from the TOBAC model in order to address NCDs more broadly. Reflecting on this model, we offer several approaches (outlined in the table below) that could bolster efforts to tackle global NCDs and inform current and future efforts to strengthen research capacity and ensure broader uptake of research results.

In addition to these approaches, the administrative architecture of the TOBAC program, like several Fogarty programs, requires that the major portion of the research funding be spent in the LMIC, which emphasizes locally relevant research and contributes to the research career pathways at LMIC institutions. These are both critical to the progress that has been made in tobacco control and will be important for addressing NCDs more broadly.

**Table:** 

Unique programmatic approaches from the TOBAC program	
Embedding capacity building into a research grant ensures that research capacity is built synergistically with research goals, allows for a high level of flexibility in models for building capacity, and enables grantees to leverage their research to train the next generation of scientists in LMICs. Specifically, it allows for:	

Research capacity building	Enhanced empirical evidence

Programs that twin HIC and LMIC scientists, thereby supporting opportunities for lasting, international collaborations; Development of scientific research career pathways and opportunities at LMIC institutions by building institutional capacity in NCD research; Continued opportunities for enhanced collaboration and communication among researchers, policy makers, and program implementers to ensure evidence is translated to policy; Capacity building in research translation and dissemination, often involving discourse at the national policy and planning levels.	An emphasis on the relevance of research to a country's needs and context; Broadening disciplinary engagement to address the cross-sectorial impacts of NCDs (economic, environmental, law, etc.); Support for implementation science research to address NCDs; Dissemination plan beyond scientific journal publication that increases the likelihood that findings will have a real-world impact.

The TOBAC program also complements work supported by other major funders. Canada's International Development Research Centre has supported tobacco control research and capacity building for over a decade, including work in tobacco economics, waterpipe smoking, and alternative crops for tobacco growers (26). Additionally, over the past 10 years, the Bill and Melinda Gates Foundation and the Bloomberg Philanthropies have devoted over $500 million to global tobacco control efforts, focused on reducing demand for tobacco products through evidence-based tobacco control policies (27, 28). These philanthropic organizations have focused primarily on supporting advocacy efforts rather than building research capacity. However, research is essential to generating support for and evaluating the impact of evidence-based policies, and, as described in the TOBAC case studies above, the TOBAC program's progress in building research capacity has contributed to policy development and implementation.

Tobacco is a risk factor for all major NCDs (i.e. cardiovascular disease, cancer, diabetes, and chronic respiratory infection). Given this, there is an important opportunity for cross-pollination and learning between these fields. The TOBAC program successfully demonstrates the ability of a relatively small number of research and research capacity grants to advance tobacco control efforts on an international scale. Learning from these evidence and research capacity efforts will go a long way toward the success of efforts to address the rising burden of NCDs in LMICs.

Confronting the global NCD crisis requires both scientific evidence and a critical mass of scientists who are well versed in regional needs. The National Institutes of Health's International Tobacco and Health Research and Capacity Building Program (TOBAC program) offers a unique and successful model for conducting empirical research and building research capacity simultaneously. The lessons learned from the TOBAC program provide a way forward for researchers, institutions, and funders to address the rise in NCDs.

## Paper context

Addressing the rising burden of NCDs in LMICs requires both empirical research and research capacity. However, research funding tends to focus on only one of these aspects. The NIH TOBAC program provides an important and unique model for simultaneously conducting research and building research capacity. This paper reviews the results of this program and highlights opportunities to expand it to address NCDs as a whole.

## Appendix A

2006–2016 NCD-related grant programs at Fogarty International Center

**Table:**

Year(s) of award	Program title
**2006**	International Clinical, Operational, and Health Services Research and Training Award (ICOHRTA)
**2008**	Millennium Promise Awards: Non-communicable Chronic Diseases Research Training Program (NCoD)
**2010**	Chronic, Non-Communicable Diseases and Disorders Across the Lifespan: Fogarty International Research Training Award (NCD Lifespan)
**The following focus on specific NCD subfields and/or on fields where NCD is likely to be a major component:**	
**2006**	International Training and Research in Environmental and Occupational Health
**2009**	Fogarty International Collaborative Trauma and Injury Research Training Program
**2011**	Brain Disorders in the Developing World: Research Across the Lifespan
**2011**	Medical Education Partnership Initiative (MEPI) Linked Awards
**2011**	International Tobacco and Health Research and Capacity Building Program
**2012**	Global Environmental and Occupational Health (GEOHealth)
**The following are not disease-specific and therefore do not exclude NCD-related projects and/or capacity building that is relevant to NCD:**	
**2008**	International Cooperative Biodiversity Groups
**2008**	Framework Programs for Global Health
**2009**	Recovery Act Limited Competition: NIH Challenge Grants in Health and Science Research
**2009**	Informatics Training for Global Health
**2010**	International Research Ethics Education and Curriculum Development Award (Bioethics)
**2010**	Recovery Act Limited Competition: NIH Director's Opportunity for Research in Five Thematic Areas
**2010**	Recovery Act Limited Competition: Program to Enhance NIH-supported Global Health Research Involving Human Subjects
**2010**	Recovery Act Limited Competition: Framework Programs for Global Health Signature Innovations Initiative
**2010**	Global Research Initiative Program for New Foreign Investigators Basic/Biomedical Science and Global Research Initiative Program for New Foreign Investigators Behavioral and Social Sciences
**2010**	International Research Scientist Development Award and Independent Scientist in Global Health Award
**2011**	Fogarty International Clinical Research Scholars and Fellows Program (FICRS-F)
**2011**	Fogarty International Research Collaboration Award Basic Biomedical and Fogarty International Research Collaboration Award Behavioral and Social Science
**2011**	Women and Girls Health Administrative Grant Supplements
**2012**	Framework Programs for Global Health Innovation

## Appendix B

2002–2012 TOBAC grants

**Table:**

Year(s) of award	Grant title	Principal investigator
**2002**	Asian Leadership Training for Tobacco Control Research	Ferry, Linda Hyder
**2002**	Cessation Research and Training in India and Indonesia	Lando, Harry Alan
**2002**	Egypt Smoking Prevention Research Initiative	Israel, Ebenezer
**2002**	Establishment of the Syrian Center for Tobacco Studies	Ward, Kenneth D.
**2002**	Mobilizing Youth for Action Against Tobacco in India	Perry, Cheryl Lee
**2002**	Monitoring tobacco mortality in 2M adults in four countries	Peto, Richard
**2002**	South Africa Adolescent Smoking: A Longitudinal Study	Brook, David William
**2002 and 2007**	Strengthening monitoring of Indian tobacco mortality	Jha, Prabhat
**2002**	Technology Assisted Dominican Republic Tobacco Control	Ossip, Deborah J.
**2002**	Tobacco Control in S. Africa: Prevention and Capacity Building	Resnicow, Ken A.
**2002 and 2007**	Tobacco Control Policy Analysis & Intervention Evaluation in China	Hu, Teh-Wei
**2002**	Tobacco Use Among Argentinean Youth: A Cohort Study	Perez-Stable, Eliseo J
**2002**	Psu-Western & Southern African Tobacco Research Project	King, Gary
**2002 and 2007**	Epidemiology & Intervention Research for Tobacco Control	Samet, Jonathan M.
**2007**	SMS Turkey: Harnessing the power of TXT messaging to promote smoking cessation	Ybarra, Michele
**2007**	The Political Economy of Tobacco Control in Southeast Asia	So, Anthony D.
**2007**	Increasing Capacity for Tobacco Research in Hungary	Foley, Kristie L.
**2007**	Advancing Cessation of Tobacco In Vulnerable Indian Tobacco consuming Youth	Reddy, Srinath K.
**2007**	Building Capacity of Tobacco Cessation in India & Indonesia	Nichter, Mark A.
**2007**	Network for Tobacco Control among Women in Parana, Brazil	Scarinci, Isabel C.
**2007 and 2012**	Responding to the changing tobacco epidemic in the Eastern Mediterranean Region	Maziak, Wasim
**2007**	Tobacco Control Research and Training in South America	Perez-Stable, Eliseo J.
**2012**	Capacity Building for Tobacco Control in Tunisia, North Africa & Middle East	Lando, Harry Alan
**2012**	Tobacco Control Network among Women in Parana, Brazil – II	Scarinci, Isabel C.
**2012**	Cinema Smoking and Youth Smoking in Latin America	Sargent, James and Thrasher, James
**2012**	Building Capacity for Tobacco Research in Romania	Foley, Kristie L.
**2012**	From Production To Retailing: Policy-Oriented Research On Tobacco Economy In Argentina	Champagne, Beatriz Marcet and Schoj, Veronica
**2012**	Tobacco Control Policy Analysis & Intervention Evaluation in China and Tanzania	Hu, Teh-Wei
**2012**	Preventing tobacco use among adolescents in Uruguay: Project Activate	Stigler, Melissa Harrell
**2012**	Building Research and Capacity on the Economic Policy-Tobacco Control Nexus in Africa	Drope, Jeffrey
